# Robot-Assisted Middle Ear Endoscopic Surgery: Preliminary Results on 37 Patients

**DOI:** 10.3389/fsurg.2021.740935

**Published:** 2021-10-06

**Authors:** Marine Veleur, Ghizlene Lahlou, Renato Torres, Hannah Daoudi, Isabelle Mosnier, Evelyne Ferrary, Olivier Sterkers, Yann Nguyen

**Affiliations:** ^1^ENT Department, Sorbonne University, AP-HP, GHU Pitié-Salpêtrière, GRC Robot and Surgery's Innovation, Paris, France; ^2^Inserm/Pasteur UMR 1120 “Innovative Technologies and Translational Therapeutics for Deafness”, Hearing Institute Paris, Paris, France

**Keywords:** PORP, TORP, cholesteatoma, tympanoplasty, safety, robotics, robot, surgery

## Abstract

**Background:** Endoscopy during middle ear surgery is advantageous for better exploration of middle ear structures. However, using an endoscope has some weaknesses as surgical gestures are performed with one hand. This may trouble surgeons accustomed to using two-handed surgery, and may affect accuracy. A robot-based holder may combine the benefits from endoscopic exposure with a two-handed technique. The purpose of this study was to assess the safety and value of an endoscope held by a teleoperated system.

**Patients and Methods:** A case series of 37 consecutive patients operated using endoscopic exposure with robot-based assistance was analyzed retrospectively. The RobOtol^®^ system (Collin, France) was teleoperated as an endoscope holder in combination with a microscope. The following data were collected: patient characteristics, etiology, procedure type, complications, mean air and bone conduction thresholds, and speech performance at 3 months postoperatively. Patients had type I (myringoplasty), II (partial ossiculoplasty), and III (total ossiculoplasty) tympanoplasties in 15, 14, and 4 cases, respectively. Three patients had partial petrosectomies for cholesteatomas extending to the petrous apex. Finally, one case underwent resection of a tympanic paraganglioma. Ambulatory procedures were performed in 25 of the 37 patients (68%).

**Results:** Complete healing with no perforation of the tympanic membrane was noted postoperatively in all patients. No complications relating to robotic manipulation occurred during surgery or postoperatively. The mean air conduction gain was 3.8 ± 12.6 dB for type I (*n* = 15), 7.9 ± 11.4 dB for type II (*n* = 14), and −0.9 ± 10.8 for type III tympanoplasties (*n* = 4), and the postoperative air-bone conduction gap was 13.8 ± 13.3 dB for type I, 19.7 ± 11.7 dB for type II and 31.6 ± 13.0 dB for type III tympanoplasty. They was no relapse of cholesteatoma or paraganglioma during the short follow-up period (<1 year).

**Conclusion:** This study indicates that robot-assisted endoscopy is a safe and trustworthy tool for several categories of middle ear procedures. It combines the benefits of endoscopic exposure with a two-handed technique in middle ear surgery. It can be used as a standalone tool for pathology limited to the middle ear cleft or in combination with a microscope in lesions extending to the mastoid or petrous apex.

## Background

Endoscopy during middle ear surgery offers several benefits over microscopic exposure as it can provide an angled view of middle ear cavities and a closer view of structures (1, 2). Surgeons may have different uses for an endoscope ranging from a simple check after cholesteatoma removal at the end of surgery to exclusive use for exposure during the entire surgical procedure (3–5). An endoscopic technique allows a trans canal, minimally invasive approach in cholesteatoma surgery with long-term results similar to techniques using a posterior approach (6). Endoscopy is particularly useful in preventing residual cholesteatoma (7). Nevertheless, this technique is not widely used despite longstanding publications by early adopters (7–9).

Classifications to better describe the exclusive use of an endoscope or in combination with a microscope have been reported to aid comparisons with traditional techniques (10). However, endoscopic surgery has some limitations as surgical gestures are performed using one hand. This may trouble surgeons accustomed to two-handed surgery. It can affect accuracy and gestures, especially in complex surgical steps requiring delicate interactions with middle ear structures (11). Middle ear surgery is performed in a reduced anatomic space using a keyhole approach and often requires constant blood suction. Even moderate bleeding can easily fill the operating field obscuring vision of critical and fragile structures such as the ossicular chain or facial nerve. Holding the endoscope means that surgeons have to choose between suction or a surgical tool in their dominant hand. This is the main reason why conventional one-handed endoscopic surgery has a lengthy learning curve (11).

To overcome this obstacle, several modified endoscope holders have been described (12–14). These devices allow double-handed surgery to be performed as in the conventional microscopic technique. Another method of holding the endoscope to assist the surgeon is to use a motorized micro-manipulator. The RobOtol^®^ system (Collin Medical, Bagneux, France) was specifically designed for middle ear microsurgery and cochlear implantation, and has been adapted to include a teleoperated endoscope holder. Its safety has been reported in a limited number of patients (15, 16). Motion of the arm bearing the endoscope is achieved by a serial kinematic chain of three perpendicular linear links at the base and three rotatory links on the distal arm. This gives six degrees of freedom, three translational and three rotational axes.

The aim of this study was to assess the safety and value of an endoscope held by a teleoperated system such as the RobOtol^®^ during middle ear surgery.

## Patients And Methods

**Figure 1:**
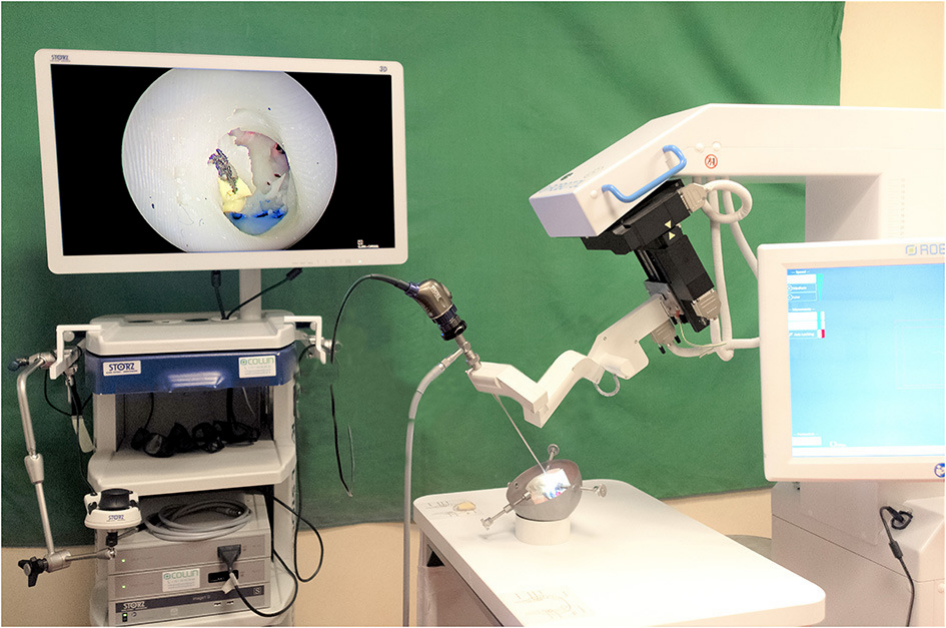
RobOtol System in endoscope holder configuration with an artificial temporal bone. The RobOtol System (Collin, Bagneux, France) is a tele-operated arm that can bear instruments or an endoscope. It can be connected to any HD camera system (Here a Image1 Storz HD endoscopy column, Karl Storz, Tuttlingen, Germany). The robot is composed of a cart bearing the arm, a display screen used to change speed and control settings. The device is driven by a space mouse (3D Connection, Waltham, MA, USA, not shown here).

**Figure 2:**
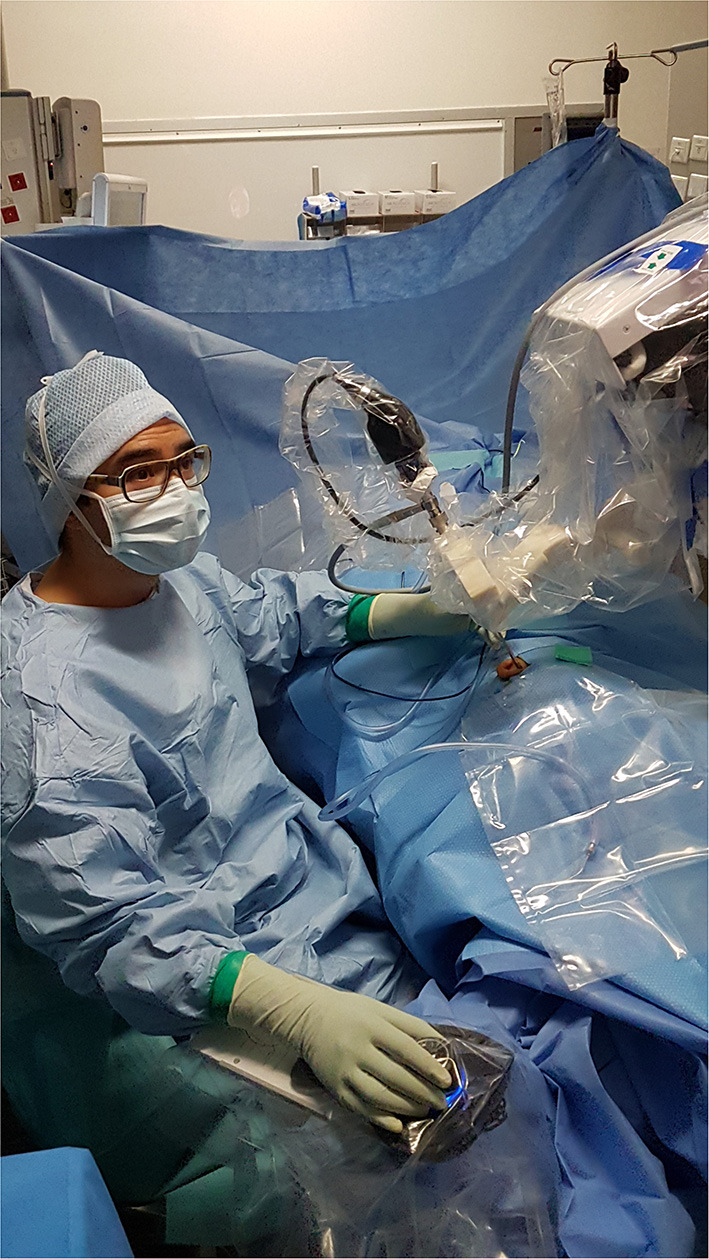
RobOtol System installed in an operating room environment. In clinical use, the robot and the endoscopy column are placed in front of the patient. The arm of the robot and the camera head are covered with a sterile drape. The device is teleoperated by a space mouse (3D Connection, Waltham, MA, USA) covered by a sterile drape.

**Figure 3:**
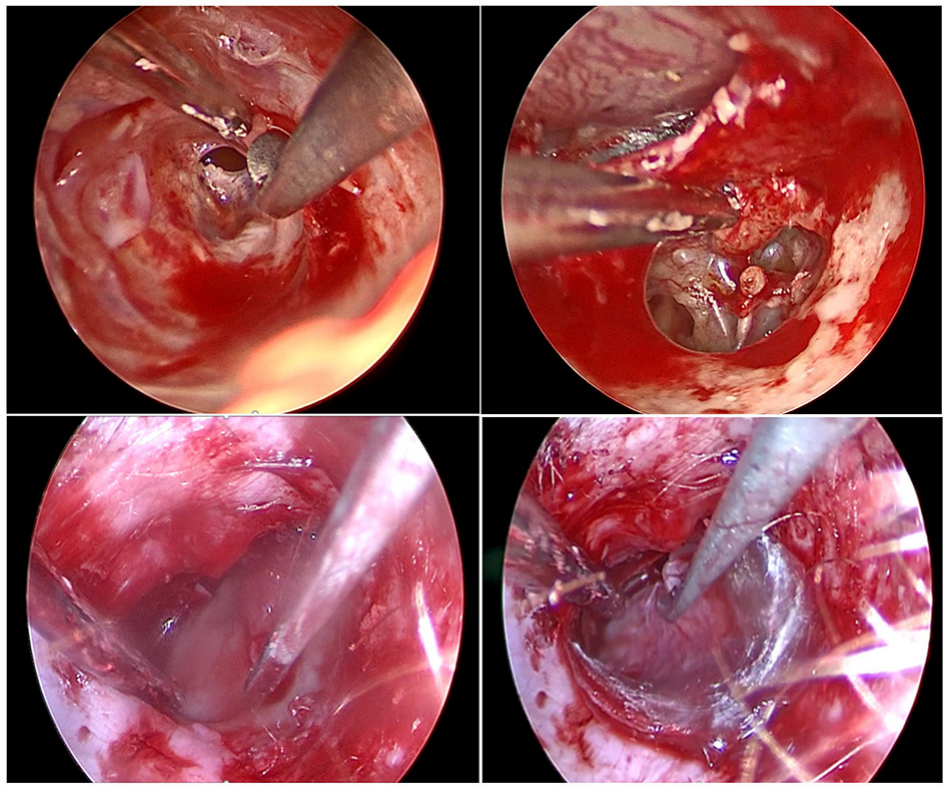
Endoscopic view of various surgical steps during middle ear surgery. Top left, Skin incision and tympano-meatal flap raising; top right, Malleus handle dissection from tympanic membrane; bottom left, cartilage grafting placement; bottom right, temporal fascia grafting placement. Note that on all picture a suction and a tool are used for a two-handed technique in combination with an endoscopic exposure.

This retrospective study included a consecutive series of patients who were treated surgically for the following pathologies: cholesteatoma, chronic otitis, tympanic perforation, retraction pocket, tympanic paraganglioma or ossicular lysis. It included cases operated on between September 2018 and February 2021. This period followed on from our previous report on our early experience (16). The surgical procedures performed were tympanoplasty type I (myringoplasty), II (partial ossiculoplasty) or III (total ossiculoplasty), revision surgical procedures for middle ear cholesteatoma or ossicular pathology, resection of tympanic paraganglioma or resection of cholesteatoma of the petrous apex. We used the RobOtol^®^ system teleoperated as an endoscope holder alone or in combination with a microscope. The system could carry 0° or 30°, 3.3 mm endoscopes (REF RBT-END-0 and REF RBT-END-30, Karl Storz, Tuttlingen, Germany). These were connected to an IMAGE1 STORZ Professional Image Enhancement System with CLARA image post-processing mode. The RobOtol system included a cart, a controller, a human–machine interface and a robot-based arm (Figure 1). We used the arm as an endoscope holder. The system is driven by the surgeon with a SpaceMouse (3Dconnexion, Waltham, MA, USA) allowing it to move the endoscope with six degrees of freedom. The robot arm is covered with a dedicated sterile drape, and a sterile adaptor provides the link between the arm and the endoscope (Figure 2). It allows to use a suction and an effector tool combined with an endoscopic exposure (Figure 3).

Our earlier study showed that the duration of set-up of the robot was 3.2–5 min and required robot cart and endoscopy column placement in the operating room, robotic arm dressing, and connection of the camera head and light source cable (16). The robot was always used in combination with a microscope as back-up, classifying all operations as two according to the EES classification (10). The duration of the operation was noted. The incision was either transcanal or retroauricular depending on the location of the cholesteatoma. The following data were collected: demographic data (age, sex), pathology, procedure type, side, previous history of otological surgery on the same side, complications during surgery, incision type, duration of surgery, and type of hospitalization (ambulatory, or conventional). Preoperative assessment included otoscopy, and pure-tone audiometry with headphones. Postoperative assessment included anatomic results with otoscopy, audiometry and complications. All patients had a preoperative audiometric test and a postoperative test 3 months after surgery. Air conduction (AC) (125–8,000 Hz) and bone conduction (BC) (250–4,000 Hz) thresholds were recorded. Thresholds at 500, 1,000, 2,000, and 4,000 Hz were used to calculate the pure-tone average (PTA) for both PTA AC and PTA BC, and the air-bone gap (ABG) as BC PTA minus AC PTA (ABG before surgery as PRE-OP ABG, ABG after surgery as POST-OP ABG). We also collected signal intensity for maximum speech intelligibility (mean “maximum speech intensity level” in dB) and mean speech recognition threshold (“SRT” in dB) before surgery and 3 months postoperatively.

Safety was defined by completion of surgery, operative time, no adverse events, and no insurmountable hindrance of visibility. All procedures performed in studies involving human participants were in accordance with the ethics standards of the institutional and/or national research committee and with the 1964 Helsinki Declaration and its later amendments or comparable ethics standards. All participants included accepted and signed a consent form to authorize data collection for this retrospective study. Data analysis was performed using the Student's *t*-test. Results are presented as mean ± standard deviation [minimum — maximum]. A *p* <0.05 was considered to indicate a statistically significant difference between groups.

## Results

This retrospective study included 37 patients (21 women, 16 men) operated on between September 2018 and February 2021. Their mean age was 41 ± 14 years old [16–71]. Pathologies were 11 cholesteatomas, eight tympanic perforations, even chronic suppurative otitis media w/o cholesteatoma, sic retraction pockets, three cholesteatomas extending to the petrous apex, one tympanic paraganglioma, and one ossicular traumatism For these pathologies, the surgical procedures were 15 type I tympanoplasties (myringoplasty), 14 type II tympanoplasties (partial ossiculoplasty), and four type III tympanoplasties (total ossiculoplasty) (Table 1). We also performed three partial petrosectomies for cholesteatomas extending to the petrous apex and one resection of a tympanic paraganglioma. Ambulatory procedures were carried out in 25 of 37 patients (68%). Twenty-two procedures used a transcanal approach (59%). There were 22 primary surgical procedures (59%). Mean duration of surgery was 155 ± 49 min [121–363 min]. Complete healing with no perforation of the tympanic membrane was noted postoperatively in all patients. No complications related to the robotic manipulation occurred during surgery or in the postoperative period. No recurrent cholesteatoma or paraganglioma was observed postoperatively but one should take into account that the follow-up period was short (<1 year on average).

**Table 1:** Demographic and pathological characteristics of the 37 cases in this study.

		***N* (%)**
Sex	Women	21 (57%)
	Men	16 (43%)
Side	Right	14 (38%)
	Left	23 (68%)
Pathology	Cholesteatoma	11 (30%)
	Chronic suppurative otitis media w/o cholesteatoma	7 (19%)
	Retraction pocket	6 (16%)
	Tympanic perforation	8 (22%)
	Ossicular traumatism	1 (3%)
	Cholesteatoma of petrous apex	3 (7%)
	Tympanic paraganglioma	1 (3%)
Surgery	Primary surgery	22 (59%)
	Revision surgery	15 (41%)
	Tympanoplasty I	15 (41%)
	Tympanoplasty II	14 (38%)
	Tympanoplasty III	4 (11%)
	Petrosectomy	3 (7%)
	Resection of tympanic paraganglioma	1 (3%)

### Tympanoplasties (Type I;II and III Results)

**Figure 4:**
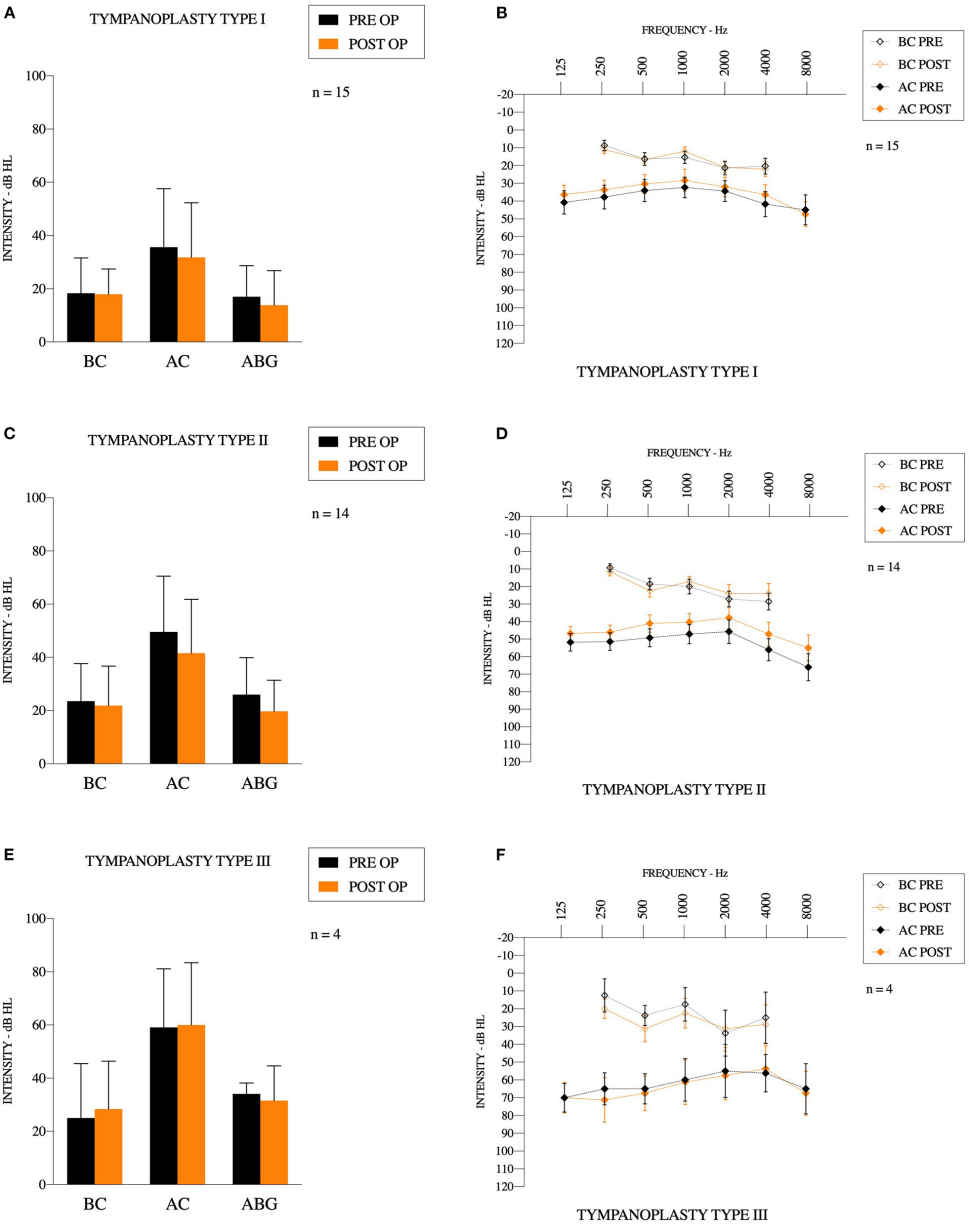
Audiometric results **(A,C,E)** results for bone conduction, air conduction and air-bone gap for Type 1,2, and 3 tympanoplasties, respectively. **(B,D,F)** Mean preoperative and postoperative audiogram for Type 1,2, and 3 tympanoplasties, respectively.

For all tympanoplasties, the pathologies were 11 cholesteatomas (33%), eight perforations (24%), seven chronic suppurative otitis media w/o cholesteatoma (21%), six retraction pockets (18%), and one ossiculoplasty (3%) (one malleus fracture with incus luxation), and Table 1. It was the first surgical procedure for 20 patients (61%). We used 22 transcanal incisions (67%) and nine posterior incisions (27%). Mean bone conduction (BC) gain was 0.5 ± 9.2 dB [−15.0–22.5], mean speech recognition threshold (SRT) gain was 4.6 ± 13.6 dB [−24.0–35.0] and mean “Max speech intensity level” gain was 2.9 ± 18.2 dB [−25.0–45.0]. All differences were not statistically significant (*p* = 0.76, *p* = 0.13, *p* = 0.67, respectively). Mean air conduction (AC) gain was 5.0 ± 11.9 dB [−21.3–38.8], with a significant improvement from preoperative to postoperative (*p* = 0.022). PRE-OP ABG was 23.0 ± 13.2 [1.3–48.8], POST-OP ABG was 18.5 ± 13.5 [1.3–51.3], again with a significant improvement from preoperative to postoperative values (*p* = 0.042). Results are reported on Figure 4.

### Group: Tympanoplasty Type I

Fifteen type 1 tympanoplasties were performed [10 women (67%)]. There were 13 ambulatory procedures (87%), 11 transcanal incisions (73%) and four posterior incisions (27%). It was the first surgery for 12 patients (80%), and revision surgery for three patients (and among these, one had multiple revisions). Mean operating time was 113 ± 32 min [51–170]. Surgery included two cholesteatomas (13%), two retraction pockets (13%), six tympanic perforations (40%), and five chronic suppurative otitis media w/o cholesteatoma (33%). Mean bone conduction gain was 0.4 ± 8.0 dB [−8.8–22.5]. Mean air conduction gain was 3.8 ± 12.6 dB [−21.3–38.8]. Mean SRT gain was 4.4 ± 7.8 dB [−8.0–18.0] and mean maximum speech intensity level was 1.4 ± 13.4 dB [−20.0–35.0]. All of these gains were not statistically significant (*p* = 0.84, *p* = 0.26, *p* = 0.06, *p* = 0.70, respectively). PRE-OP ABG was 17.3 ± 11.7 [1.3–38.8], POST-OP ABG was 13.8 ± 13.3 [1.3–51.3], and the difference was not statistically significant (*p* = 0.13).

### Group: Tympanoplasty Type II

Fourteen type II tympanoplasties were performed on six women (43%). There were 10 ambulatory procedures (71%), and eight transcanal incisions (57%). It was the first surgery for six patients (43%), and revision surgery for eight patients (and among these, four had multiple revisions). Mean operating time was 148 ± 38 min [97–225]. Surgery included eight cholesteatomas (57%), one traumatism (incus luxation) (7%), three retraction pockets (21%), and two perforations (14%). In seven cases (50%), we used a titanium partial ossicular prosthesis (Heinz Kurz GmbH Medizintechnik, Dusslingen, Germany). The size of the prosthesis was 2.8 ± 0.2 mm [2.5–3.0]. In five cases (36%), we used Otomimix bone cement (Olympus, Hamburg, Germany). In one case (7%), we removed fibrosis around a partial prosthesis. In one case (7%), we used cartilage to perform the ossiculoplasty. Mean air conduction gain was 7.9 ± 11.4 dB [−6.3–36.3] and the difference between preoperative and postoperative values was significant (*p* < 0.05). Mean bone conduction variation was 1.7 ± 11.3 dB [−15.0–21.3], mean SRT gain was 4.6 ± 19.0 dB [−24.0–35.0] and mean maximum speech intensity level gain was 5.0 ± 23.2 dB [−25.0–45.0]. All of these gains were not statistically significant (*p* = 0.58, *p* = 0.69, *p* = 0.79, respectively). PRE-OP ABG was 26.0 ± 13.9 [6.3–48.8], POST-OP ABG was 19.7 ± 11.7 [6.3–40.0], and the difference was statistically significant (*p* = 0.003).

### Group: Tympanoplasty Type III

Four type III tympanoplasties were performed on three women (75%) and a man (25%). There were two ambulatory procedures (50%), and three transcanal incisions (75%). It was the first surgery for two patients (50%), and revision surgery for two patients (multiple revisions). Mean operating time was 153 ± 52 min [77–195]. Surgery included one cholesteatoma (25%), one retraction pocket (25%), and one chronic otitis (50%). In all cases, we used a titanium total ossicular prosthesis (Heinz Kurz GmbH Medizintechnik, Dusslingen, Germany). The size of the prosthesis was 4.4 ± 0.7 mm [4.0–5.5]. Mean bone conduction gain was −3.4 ± 5.6 dB [−11.3–1.3]. Mean air conduction gain was −0.9 ± 10.8 dB [−13.3–12.5]. Mean SRT gain was 5.5 ± 11.9 dB [−2.0–23.0] and mean max speech intensity level gain was 1.3 ± 19.3 dB [−10.0–30.0]. PRE-OP ABG was 34.1 ± 4.1 [30.0–38.8], and POST-OP ABG was 31.6 ± 13.0 [15.0–43.8]. All of these gains were not statistically significant (*p* = 0.84, *p* = 0.26, *p* = 0.06, *p* = 0.70, *p* = 0.74, respectively).

### Group: Cholesteatoma of the Petrous Apex

We performed three surgical procedures on the petrous apex of one woman (33%) and two men (66%) and none were ambulatory procedures. We only used posterior incisions. It was the first surgery for one patient and the others already had multiple surgical procedures. Mean operating time for the three procedures was 181 ± 100 min [100–293].

### Group Resection of Tympanic Paraganglioma

One resection of tympanic paraganglioma was performed on one woman, with conventional hospitalization and with a retroauricular incision. The operating time was 84 min and it was a primary procedure.

## Discussion

Middle ear surgery has always been a highly specialized surgical procedure. It requires lengthy training due to the confined surgical space, the risk of injury to sensorineural structures requiring excellent anatomic knowledge, and the intense practice that is required. Endoscopes have shown some benefits over the microscope in different aspect of this surgery (9), with decreased morbidity for second-look procedures, enhanced visualization including a wider angle of view and reduced operating time (17, 18). Difficult exposure of middle ear recesses demanding extensive drilling is one of the major reasons for residual disease, particularly for hidden structures such as the sinus tympani, anterior epitympanic recess, and eustachian tube as these areas are considered to be at risk of cholesteatoma recurrence (7). Indications and popularity have been steadily increasing and endoscope holders have been described, confirming the efficacy of two-handed endoscopic surgery (12–14).

In this study, the first aim was to demonstrate the safety and feasibility of endoscopic robot-assisted surgery in various middle ear operations such as type I, II or III tympanoplasties, cholesteatoma of the petrous apex, and tympanic paraganglioma. In this study, all patients benefited from complete healing with no perforation of the tympanic membrane and no complications related to the robotic manipulation either during surgery or in the postoperative period. All procedures were completed, and the robotic arm did not interfere with exposure of the surgical field, and was able to expose every middle ear location. For type I, II, and III tympanoplasties, the first aim was to close the tympanic membrane, and improve the air conduction (or obtain stable air conduction) without changes in bone conduction.

We have previously reported our surgical results for partial ossicular replacement prosthesis (PORP) and total ossicular replacement prosthesis (TORP) ossiculoplasties performed with a microscopic technique and using titanium prostheses (19); success was defined as a postoperative ABG ≤ 20 dB. At 2 months postoperatively, surgical success was achieved in 66% of the PORP group and 49% of the TORP group in that earlier series (19). The results in the present series were 57% success in the PORP group and 25% success in the TORP group. This can be explained by the fact that the main operator had no previous experience of endoscopic surgery and proceeded straight to robot-based endoscopic surgery. Previous reports comparing one-handed endoscopic surgery with microscopic surgery showed that audiometric results were similar in both techniques and we expect the audiometric results to be similar once the learning curve is overcome (1, 20). Future prospective studies will have to be conducted to compare our results with microscopic or robot-based technique. Comparison of robot-based technique will also have to be performed by groups who have more experience than we for one-handed ear endoscopic surgery.

For cholesteatoma of the petrous apex and tympanic paraganglioma, the aim was to achieve no relapse of the pathology, and, so far, this is the case for every patient in this study (at least on 1-year postoperative MRI, but this result needs to be confirmed in a longer follow-up period). In cholesteatoma cases, we were able to see every hidden structure in the middle ear, even behind the jugular bulb in extended lesions. In tympanic paraganglioma, it was easier to control the retraction of the paraganglioma during laser treatment and localize its vascular pedicles. The mean operating time was acceptable in all procedures, and preparation of the robot was performed during patient anesthesia induction to reduce the duration of installation. Clearly, robot-assisted endoscopy does not erase every disadvantage of endoscopes such as loss of depth of perception and binocular vision. But using a robot removes the disadvantage of a one-handed surgical technique, allows suction to control bleeding, and reduces vapor through constant replacement of the air in the external auditory canal (this could be further improved by humidifying the tip of the endoscope with diluted soap). If condensation or blood hinders endoscopic vision, instead of removing the endoscope from the external auditory canal, the surgical field could be washed out with saline serum. Endoscope cleaning was not time-consuming.

Compared to the other endoscope holders, the robot-based holder offers a more accurate control with tremor suppression. In the future, upgrades such as contact or collision detection or coupling with navigation system and augmented reality can also be envisioned. In the future, we could also implement a video based instruments automated tracking and the robot could drive the endoscope to follow the instrument all along. This can be done a surgical assistant today. On the other hand, the robot-based technique is less dynamic than a conventional endoscopic technique. Placing in and removing the endoscope is faster with a manual technique and dynamic constant changes of endoscope position may help the operator to obtain a depth perception of the anatomical structure that is impaired with an endoscope.

In this series, the duration of surgery was not reduced compared to a microscopic technique. Learning curve analysis with cumulative summation test for learning curve (LC/CUSUM) needs to be performed to check if the surgeons had reached the necessary skill plateau. Pan et al. have shown that neuro-endoscopes (such as those used with the RobOtol system) cause higher thermal release in the surgical cavity so it is important to apply submaximal light intensity. For the whole procedure, LEDs should be used at submaximal intensity and the operating field should be regularly rinsed (21).

**Figure 5:**
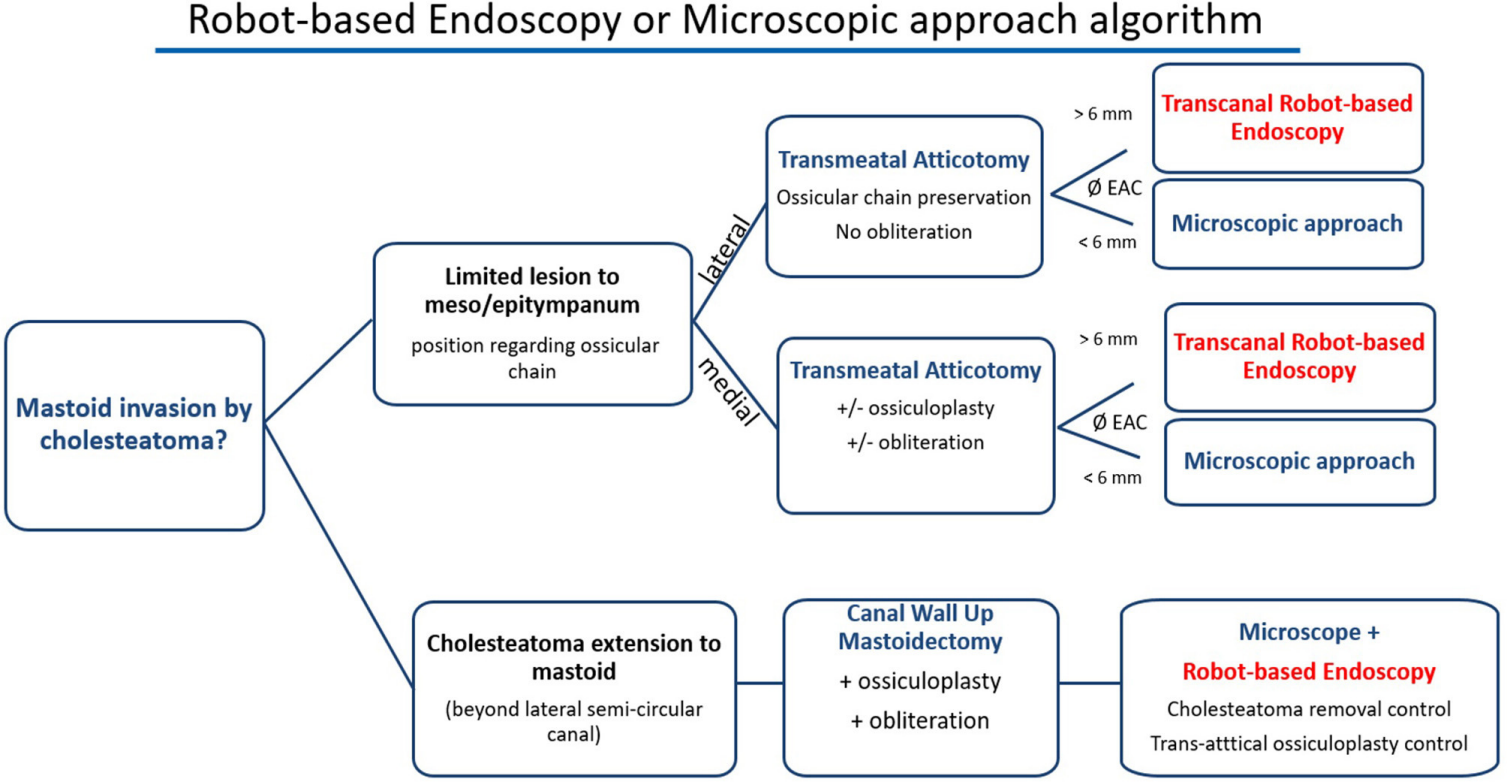
Proposed algorithm for robot-based endoscopy indication. Robot-based endoscopy should be used as an additional tool to the microscope and not as a competing device. Its main limitation is the external auditory canal (EAC) diameter that may limit the use of two tools and the endoscope. We propose the following decision tree to choose the use of the robot-based endoscopy, the microscope and both to perform the approach and surgery.

The main limitation of the technique that we encountered was the external auditory canal diameter. We did not systematically measure its size but it was difficult to perform an exclusive endoscopic approach when a 6-mm diameter speculum could not fit into the external auditory canal. In the case of a narrow canal, we experienced collisions between the two tools and the endoscope limited access with the tool in some anatomic regions. This could be resolved by maintaining the endoscope further from the middle ear cleft in the canal but this reduced the quality of exposure. This may limit the use of robot-based endoscopy in children. Therefore, we would not recommend the use of a two-handed technique and a 3.3-mm diameter robot-based endoscope if the external auditory canal is <6 mm wide (Figure 5). This limitation will be eliminated in the near future as narrower endoscopes are currently being developed for the RobOtol system.

## Conclusion

This study indicates that robot-assisted endoscopy is a safe and trustworthy tool for several categories of middle ear procedures. It combines the benefits of endoscopic exposure with a two-handed technique in surgery of the middle ear. It can be used as a standalone tool for pathology limited to the middle ear cleft or in combination with a microscope in lesions extending to the mastoid or petrous apex. The RobOtol system can be used safely and with accurate control as an endoscope holder. The next step will be to compare robot-assisted endoscopy with conventional microscope surgery.

## Data Availability Statement

The raw data supporting the conclusions of this article will be made available by the authors, without undue reservation.

## Ethics Statement

The studies involving human participants were reviewed and approved by institutional review board (CNIL National Committee for data protection number 20191219182243). The patients/participants provided their written informed consent to participate in this study.

## Author Contributions

MV and YN collected the data and wrote the article. EF and OS contributed to the design and implementation of the research. GL, HD, and RT revised the manuscript and contributed to data analysis. All authors contributed to the article and approved the submitted version.

## Funding

The study was supported by research funding from Fondation pour l'Audition (Starting Grant IDA-2020) and ANR- 18-CE19-0005 (Murocs).

## Conflict of Interest

YN and OS are consultants for Collin Medical. The remaining authors declare that the research was conducted in the absence of any commercial or financial relationships that could be construed as a potential conflict of interest.

## Publisher's Note

All claims expressed in this article are solely those of the authors and do not necessarily represent those of their affiliated organizations, or those of the publisher, the editors and the reviewers. Any product that may be evaluated in this article, or claim that may be made by its manufacturer, is not guaranteed or endorsed by the publisher.
